# Azure B as a novel cyanide antidote: Preclinical in-vivo studies

**DOI:** 10.1016/j.toxrep.2020.10.015

**Published:** 2020-10-20

**Authors:** Philippe Haouzi, Marissa McCann, Nicole Tubbs

**Affiliations:** Division of Pulmonary and Critical Care Medicine, Department of Medicine, Pennsylvania State University, College of Medicine, Hershey, PA, USA

**Keywords:** Cyanide, Antidote, Blue dyes

## Abstract

We have determined the effects of azure B (AzB), the main demethylated metabolite of methylene blue (MB), on a model of lethal cyanide intoxication. Our rationale was the following: AzB 1- possesses redox properties very similar to those of MB, which is a potent cyanide antidote, 2- may present a higher intracellular diffusibility than MB, 3- is already present in commercially available solutions of MB, and 4- appears very quickly in the blood after MB administration. AzB could therefore be a member of the phenothiazium chromophore family of interest to treat cyanide intoxication. We found, in spontaneously breathing urethane sedated rats, that AzB mimicked the effects of MB by increasing metabolism, ventilation and cardiac contractility up to 30−40 mg/kg. AzB had a lethal toxicity when the dose of 60 mg/kg was reached. Doses of AzB were therefore chosen in keeping with these data and the doses of MB previously used against cyanide intoxication (4–20 mg/kg) in the rat – doses corresponding to those used in humans to treat methemoglobinemia. KCN, infused at the rate of 0.375 mg/kg/min iv for 13 min, was fatal within 15 min in 100 % of our un-anesthetized rats. AzB at the dose of 4 mg/kg (n = 5) or 10 mg/kg (n = 5) administered 3 min into cyanide infusion allowed 100 % of the animals to survive with no clinical sequelae. The onset of coma was also significantly delayed and no apnea or gasping occurred. At the dose of 20 mg/kg, AzB was much less effective.

At 4 mg/kg, the antidotal effects of AzB were significantly better than those produced by MB at the same dose and were not different from the effects produced by 20 mg/kg MB. We conclude that AzB is a potent cyanide antidote at relatively low doses.

## Highlights

• Azure B at 4 mg/kg is an extremely potent Cyanide antidote in unsedated rats.
• Azure B was very effective in preventing apnea as well as gasping and cardiac arrest in lethal cyanide intoxication.
• Azure B appears to be more effective than Methylene blue at low doses.

## 1 Introduction

Cyanide, one of the most emblematic mitochondrial poisons, represents a persisting criminal, terrorist [4] and accidental threat [1,5,12,23,46]. The acute and early depressive effects of relativity low levels of cyanide on cardiomyocyte contractility [2,24,25,38,50] and on the activity of the medullary respiratory neurons [17,37] are immediately life-threatening [6,26].

We have recently reinvestigated the antidotal effects of methylene blue (MB), which was briefly considered in the early 20th century [13,27,53] as a treatment of acute cyanide intoxication. We found that MB is very effective against lethal and sublethal cyanide intoxication [20,32,33]. MB at 4, 10 or 20 mg/kg, corresponding to the doses currently used in humans for the treatment of methemoglobinemia (∼ 0.5–2.5 mg/kg) [55], allowed all rats intoxicated by cyanide to survive, while preventing cyanide induced apnea *in a dose dependent manner* [33]. These antidotal properties could be explained by MB redox properties, allowing a direct rescue of the Krebs (TCA) cycle (NADH oxidation) and of the mitochondrial membrane potential. An increased turn-over of the formation of oxidized hemoglobin in red cells, in turn trapping free cyanide, as well as of the metallo-proteins of the electron chain [18,20,31,32] could also be involved. MB appears to have an even better efficacy than hydroxocobalamin [32]. These results fit well with other studies that support the view that mitochondrial-directed therapy can, in contrast to hydroxocobalamin, mitigates mitochondrial dysfunction imposed by cyanide [43]. Using an agent, such as MB, that is capable of rapidly opposing the deleterious metabolic consequences of cyanide intoxication, certainly represents a unique approach to treat cyanide intoxications [4,6,9], as well as other mitochondrial poisons [34,39,48,49]. Indeed, the treatment of an acute cyanide intoxication currently relies on families of antidotes [4,6,9] aimed at decreasing the concentration of exchangeable cyanide in the blood and tissues, by either trapping free cyanide [3,7,8,10,15,16,42] or enhancing its elimination in a non-toxic form [7,[28], [29], [30],^44^,^45^]. None of these agents have a direct effect of cellular metabolism.

The question addressed in the present study is whether azure B (AzB), the main demethylated metabolite of MB [52], possesses antidotal properties in a model of lethal cyanide intoxication in un-anesthetized rats. Of interest, AzB alone has been shown to have a *higher potency than MB* in various life-threatening conditions. For instance, Culo et al. [22] found that AzB could rescue 10 out of 10 mice from experimentally-induced endotoxic shock, versus 2 animals out of 10 for MB. AzB also reduces the inhibition of glutathione reductase [14] and tau-protein aggregation [54] at significantly lower concentrations than MB. We have recently found that while AzB should produce redox and metabolic effects similar to MB, its administration led to higher levels of blood pressure and metabolism as compared to MB in sulfide-intoxicated rats [49]. No beneficial effects were observed after administration of thionine, the completely demethylated metabolite of MB [49]. Of note, the distinction between the effects of MB and those of AzB has seldom been considered in the literature [47], while AzB is often present in commercial solutions of MB and appears rapidly in the blood after MB administration [40].

One reason for a potential higher efficacy of AzB than MB could be that AzB is neutral in its quinoneimine form and thus may have a faster/better diffusion through cellular and mitochondrial membranes than MB, which is positively charged [51]. Such a property may prove to be crucial during life-threatening cyanide intoxication. Finally, AzB has already been administered in rodents [22], and even orally in humans [47], showing kinetics very similar to those of MB, as illustrated by Kim et al. [40].

In this study, we have first established, in control rats, the dose response relationship and toxicity of AzB on respiration, metabolism and circulation. Then, in keeping with this toxicity study, we have determined whether AzB can counteract cyanide induced coma, seizure, apnea/gasping and cardiac arrest in awake rats following exposure to a lethal infusion of cyanide [33]. The doses of AzB that we used were similar to those found to be effective with MB.

## 2 Materials and methods

The Penn State Hershey Medical Center Institutional Animal Care and Use Committee approved the study. The rats were housed at the Animal Resource Services at the Penn State College of Medicine, which conforms to the requirements of the US Department of Agriculture and the Department of Health, Education and Welfare.

### 2.1 Characterization of AzB solution

**Fig. 1:**
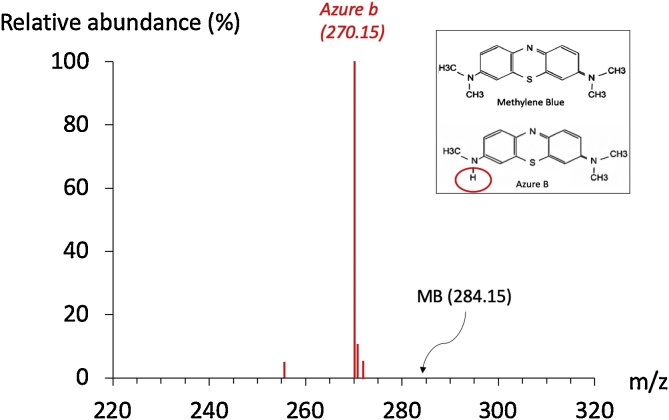
Spectrum of the solution of azure B used in the present study. The peak at *m/z* = 270 corresponds to azure B. Samples of the powder dye were weighed and dissolved in LCMS-grade methanol at a concentration of 1 mg/mL. Note that the solutions that we used were almost exclusively composed of AzB.

The solutions of AzB (*N,N,N′*-Trimethylthionin, Sigma-Aldrich # A4043) that we used for the experiments were analyzed by mass spectrometry (Waters Q-TOF Premier quadrupole/time-of-flight (TOF) mass spectrometer). The compound was essentially a salt, the positive ion portion of the compound was observed, making the counter ions (Cl^−^) “invisible” to the mass spectrometer. We found that our solutions were almost exclusively composed of AzB (3 methyl groups) (Fig. 1).

### 2.2 Effects of incremental dose of AzB in spontaneously breathing urethane anesthetized rats

Animals were anesthetized by an intra-peritoneal injection of urethane (1.8 g/kg) as previously described [36]. The rats were tracheostomized and spontaneous inspiratory flow was measured using a pneumotachograph (Series 1100, Hans Rudolph, Inc. Shawnee, KS) connected to the tracheostomy via small dead space two-way valve. Mixed expiratory gas composition was continuously analyzed (Gemini,CWE Inc.,USA) from a 7 mL mixing chamber in series with the expiratory side of the valve, and oxygen uptake (V̇O_2_) was computed as previously described [36].

A catheter (PE-50) was placed into the right femoral artery to continuously monitor arterial blood pressure (ABP) and for sampling arterial blood. The arterial catheter was connected to a pressure transducer (MLT844 Physiological Pressure Transducer, AD Instruments, Colorado Springs CO). A similar catheter was placed into the left jugular veins, for injecting AzB, and another one in a carotid artery. The carotid catheter was advanced caudally and its tip was placed in the left ventricle. Left ventricular systolic pressure (LVSP) and heart rate (HR) were determined.

Arterial blood pressure, mixed expired O_2_ and CO_2_ fractions, and respiratory flow signals were also fed into a PowerLab/chart system (AD Instruments, Colorado Springs CO) for the computation of minute ventilation and O_2_ consumption ($\dot{\text{V}}$ O_2_); dP/dt max, a marker of cardiac contractility, was computed as the peak of the first derivative of the left ventricular pressure signal as a function of time. Lactate levels were measured using an i-STAT1 blood gas analyzer (ABAXIS, Union City, CA). All signals were digitized visualized on-line. Data were stored for further analysis using LabChart7. Arterial blood was sampled for measurement of lactate every 5 min.

### 2.3 Effects of AzB following lethal cyanide intoxication in awake animals

The model and protocol have been previously described in detail [33]. Potassium Cyanide (Sigma, ref 60,178) solution were prepared at the concentration of 0.5 mg/mL diluted in sterile saline [33,36]. We have previously established that an infusion of potassium chloride at the same concentrations of potassium cyanide had no measurable effects on circulation or breathing and did not produce any neurological effect [36]. Azure B (AzB, 10 mg/mL) was diluted in sterile saline to maintain constant the total volume that was administered (2 mL) (see next paragraph for the doses of AzB).

### 2.4 Protocol and data analysis

On the day of the study, a heparinized, double lumen, venous catheter was placed in the dorsal vein of the tail as previously described [33]. At least 2 h were given for full recovery before doing the study. Potassium cyanide was infused intravenously at the rate of 0.375 mg/kg/min for 13 min (780 s). This exposure leads to a lethal cyanide intoxication: following a short phase of locomotor agitation, a coma associated with seizures developed within minutes; followed by a period of apneas and leading to a cardiac arrest (CA). CA was defined by the disappearance of the perception of cardiac pulsation on the chest and complete whitening of the pupils [33].

AzB was infused 3 min (180 s) after the onset of cyanide infusion, when the animal displayed signs of ataxia. Three different doses of AzB were used in keeping with the dose effect relationship obtained in control rats. As developed in the result section the chosen doses were 4, 10 and 20 mg/kg that were infused for 5 min (300 s). Animals were monitored every 15 s for 20 min, then every minute for the following hour in the surviving rats. All surviving animals were examined and weighed every day for 2 weeks. Our primary outcomes were the onset of coma, seizures, apnea/gasping, asystole along with the duration of coma in the animals that survived the intoxication. Data are presented as median and range. Comparisons between groups (control and treated at any given dose) were done using non-parametric statistics (Mann Whitney U test for 2 group comparisons). Statistical analyses were done with GraphPad Prism 6 (Graphpad Software, La Jolla, CA). P < 0.05 was regarded as significant for any of these comparisons.

## 3 Results

### 3.1 Metabolic and circulatory effects of AzB infusion in spontaneously breathing urethane sedated rats

**Fig. 2:**
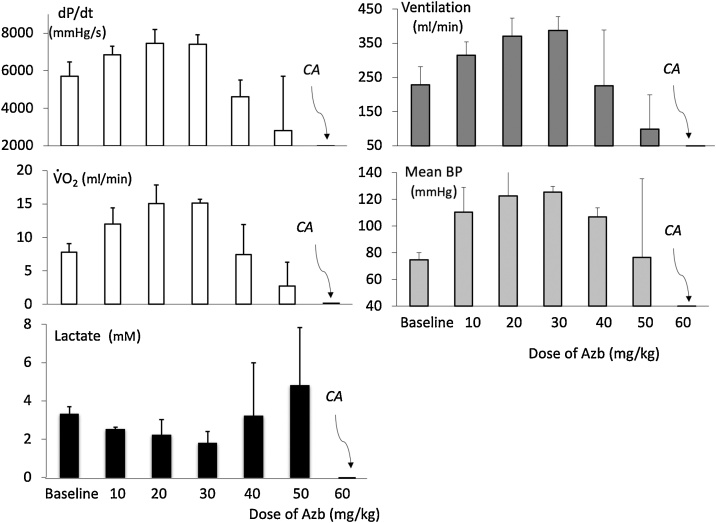
Effects of a ramp-like infusion of AzB on left ventricular contractility (dP/dt max), $\dot{\text{V}}$ O_2_, minute ventilation, mean blood pressure and lactate levels in 6 sedated rats. Data are shown as mean ± SD. AzB increased O_2_ consumption along with ventilation, blood pressure and cardiac function up to 30 mg/kg. Above 30-40 mg/kg there was a progressive deterioration of the cardiac function with a depression in breathing along with an increase in lactate levels, leading to death at ∼ 60 mg/kg.

AzB was infused in 6 Sprague-Dawley rats, weighing 437 ± 60 g, at the rate of 2 mg/kg/min until death occurred. AzB administration increased $\dot{\text{V}}$ O_2_, minute ventilation, mean arterial blood pressure and left ventricular dP/dt max up to a dose of 30 mg/kg (Fig. 2). Above 30 mg/kg, all these variables started to subside, along with a rise in lactate concentration, leading to death, at the dose of 60 mg/kg, by apnea and asystole (Fig. 2).

### 3.2 Cyanide intoxication in unsedated rats

**Fig. 3:**
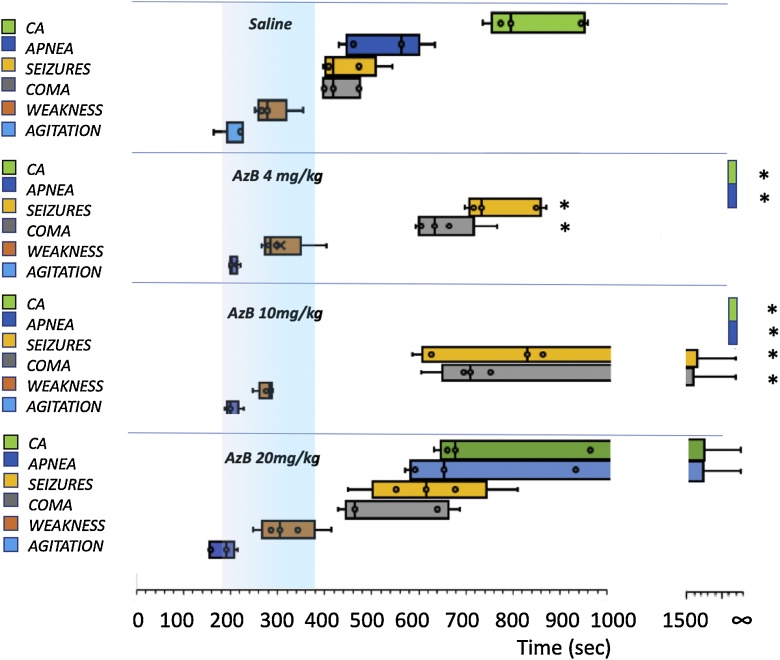
Effects of the different doses of AzB vs saline, infused 3 min into cyanide exposure, on the symptoms of cyanide toxicity. Time 0 is the onset of cyanide administration; the blue area corresponds to the period of saline or AzB injection. Boxes display the median and range of the time of the onset of the toxic effects of cyanide. Data are shown for each dose of AzB. The effects of AzB were similar at 4 mg/kg and 10 mg/kg: In contrast to the saline group, none of the animals presented an apnea and they all survived. The onset of coma was significantly delayed at either dose. Following 20 mg/kg, the effects of AzB were not significantly different from untreated intoxication. * p < 0.01, untreated vs AzB, Mann Whitney U test. CA: cardiac arrest.

A total of 20 male Sprague-Dawley rats (Charles River Laboratories), weighing 384 ± 33 g, were studied. The effects of infusion of cyanide (n = 5) were very similar to those we have previously reported in a larger number of rats [33]. The animals displayed a phase of locomotor hyperactivity 212 s (median) into cyanide infusion, followed by difficulty walking, which led to a coma and seizures at 404 s. All of the rats presented an apnea associated with a gasping respiratory pattern 548 s into cyanide exposure, followed by a cardiac arrest at 782 s (Fig. 3).

### 3.3 Effects of Azb 4 mg/kg (n = 5), 10 mg/kg (n = 5) and 20 mg/kg (n = 5)

The effects of the different doses of AzB are shown in Fig. 3. Responses were similar at 4 mg/kg (n = 5) and 10 mg/kg (n = 5) and consisted in the survival of all animals and a dramatic delay in the occurrence of neurological outcomes. Indeed, whether 4 or 10 mg/kg were used none of the treated animals presented an apnea or a gasping pattern, reflecting the absence of depression of medullary respiratory neurons at either dose. Of note, the median of the duration of coma was 746 s and 373 s for 4 and 10 mg/kg AzB, respectively, whereas all the control animals expired and never recovered from their coma (p < 0.01). All of the animals that received 4 or 10 mg/kg AzB had no difficulty with feeding nor did they display any abnormal behavior after 24 h following the intoxication. The antidotal effects of AzB at the dose of 20 mg/kg were blunted and although seizures and apnea onset were delayed in some rats (Fig. 3), four out of five rats still died with the same time course as the control animals.

## 4 Discussion

In control sedated animals, AzB at dose up to 30 mg/kg, just like MB [31,32], increased $\dot{\text{V}}$ O_2_. As developed in our previous mechanistic studies [31,32,35]. Such an increase in $\dot{\text{V}}$ O_2_ should not be considered as resulting from a cellular hypermetabolism, but is the direct re-oxidation of leucoMB by oxygen [31,32,35]. AzB also produced the exact same effect as MB, on minute ventilation and cardiac contractility [31,32]. We found that at a dose of 4 mg/kg, well below its level of toxicity, AzB is a very effective antidote against a rapidly lethal form of cyanide intoxication. In cyanide intoxicated rats, the toxicity of AzB seems to counteract its antidotal properties at 20 mg/kg. AzB toxicity consisted in rapid depression in cardiac contractility (dP/dt max) and breathing leading to cardiac arrest, a phase that was preceded by an increase in the concentrations of lactate in the blood.

### 4.1 Model of KCN intoxication in the rat

We used a rat model of intravenous exposure to cyanide, a model that we have previously validated [36], which we found to be more reproducible than following intra-peritoneal injections [11,21,36]. This model recapitulates in a predictable manner the systemic symptoms of lethal cyanide intoxication (agitation, motor weakness, coma and seizure rapidly followed by a gasping pattern of breathing, a central apnea and a rapid cardiac arrest). All of the non-treated (saline) unsedated animals died by cardiac arrest following the infusion of ∼ 5 mg/kg of cyanide (administered over 13 min). Although these exposures may not replicate the toxicity on the lungs of cyanide inhalation, they represent reproducible, easy to handle and to calibrate models of non-inhaled lethal KCN intoxication [36].

### 4.2 Treatment of life-threatening cyanide intoxication by AzB

We have previously demonstrated that MB could be used against lethal sulfide and cyanide intoxications [19,39,48]. AzB, just like MB, possesses very distinctive cyclic redox properties, which greatly impact the effects of cyanide intoxication [32]. AzB can be readily reduced into their leuco-form by NADH before being re-oxidized by O_2,_ giving electrons in the process, allowing for a new cycle of reduction [14]. By analogy with the couple MB/LeucoMB, the redox couple AzB/LeucoAzB could restore the TCA cycle and the glycolytic activity by oxidizing NADH and decreasing the NADH/NAD ratio [41]. Whenever NADH is unable to be oxidized by the mitochondrial complex I, like during cyanide intoxication for instance, some ATP could still continue to be produced via succinyl-CoA synthase, i.e. via *mitochondrial substrate-level phosphorylation* [41].

### 4.3 AzB versus MB

**Fig. 4:**
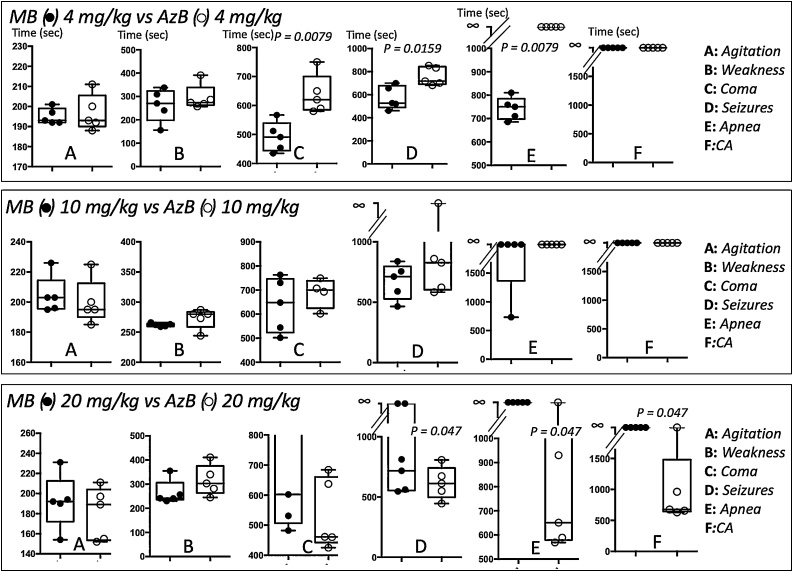
Comparison of the effects of the different doses of AzB to those of MB that we have previously reported in the very same model of cyanide intoxication [33]. AzB at the dose of 4 mg/kg was much more effective than MB on the onset of coma, seizures or apnea, while AzB efficacy was counterbalanced by its toxicity at 20 mg/kg, which represented the optimal effective dose for MB [33]. Note that 4 mg/kg AzB was as effective as 20 mg/kg MB. P values are reported for each comparison only when significant (p < 0.05. Mann Whitney U test).

We have previously found that the maximal antidotal effects of MB were observed at 20 mg/kg, while at 4 mg/kg these effects were much weaker [33]. We have compared in Fig. 4 the effects of MB that were previously obtained in the very same model of cyanide intoxication [33] to those of AzB, reported in the present study. At the dose of 4 mg/kg, AzB was significantly more effective than MB. Also, 4 mg/kg AzB and 10 or 20 mg/kg MB produced similar antidotal properties against the neurological manifestations of cyanide. As mentioned in the introduction, this “superiority” of AzB at the lowest doses could be accounted for by the neutral quinoneimine form of AzB, allowing for a faster and easier diffusion of AzB inside cells than the positively charged MB [51]. This mechanism could also explain that the toxicity of AzB (Fig. 2) develops at doses lower than those previously established for MB (60 mg/kg) [33]. The present results also suggest that azure B formed in the blood and tissues, after MB administration, could be an unrecognized contributor to the antidotal effects of MB. In conclusion, AzB could be an interesting candidate in the phenothiazium chromophore family against cyanide intoxication to be considered when low doses/volumes are to be administered.

## Declaration of Competing Interest

The authors declare that they have no known competing financial interests or personal relationships that could have appeared to influence the work reported in this paper.
